# Population pharmacokinetics of azithromycin and chloroquine in healthy adults and paediatric malaria subjects following oral administration of fixed-dose azithromycin and chloroquine combination tablets

**DOI:** 10.1186/1475-2875-13-36

**Published:** 2014-01-29

**Authors:** Qinying Zhao, Thomas G Tensfeldt, Richa Chandra, Diane R Mould

**Affiliations:** 1 Pfizer Inc, 445 Eastern Point Road, MS8260-2112, Groton CT 06340, USA; 2 Novartis, Cambridge, MA, USA; 3 Projections Research Inc, Phoenixville, PA, USA

**Keywords:** Population pharmacokinetics, Malaria, Azithromycin, Chloroquine

## Abstract

**Background:**

Population pharmacokinetics (PK) of azithromycin (AZ) and chloroquine (CQ) following administration of fixed-dose combination tablet formulations of AZ and CQ (AZCQ) was evaluated using data from two studies: 1) in children with symptomatic uncomplicated falciparum malaria in sub-Saharan Africa; and 2) in healthy adults in the United States.

**Methods:**

Study 1 included paediatric subjects randomized to either AZCQ or artemether-lumefantrine treatment in Cohort 1 (age 5–12 years) and Cohort 2 (age 6–59 months). Dosing of AZCQ was approximately 30 mg/kg AZ and 10 mg/kg CQ once daily for 3 days (for ≥20 kg weight: AZ/CQ 300/100 mg per tablet; 5 to <20 kg weight: AZ/CQ 150/50 mg per tablet). Study 2 included adults randomized to receive either two AZCQ tablets (AZ/CQ 250/155 mg per tablet) or individual commercial tablets of AZ 500 mg and CQ 300 mg. Serum AZ and plasma CQ concentrations from both studies were pooled. Population PK models were constructed using standard approaches to evaluate the concentration-time data for AZ and CQ and to identify any covariates predictive of PK behaviour.

**Results:**

A three-compartment PK model with linear clearance and absorption adequately described AZ data, while a two-compartment model with linear clearance and absorption and an absorption lag adequately described CQ data. No overall bias or substantial model misspecification was evident using diagnostic plots and visual predictive checks. Body weight as an allometric function was the only covariate in the final AZ and CQ PK models. There were significantly lower AZ (0.488 vs 0.745 [mg•h/L]/[mg/kg], p < 0.00001) and CQ (0.836 vs 1.27 [mg•h/L]/[mg/kg], p < 0.00001) exposures (AUC_inf_) normalized by dose (mg/kg) in children compared with the adults.

**Conclusions:**

The PK of AZ and CQ following administration of AZCQ was well described using a three- and two-compartment model, respectively. AZ and CQ exhibited linear absorption and clearance; the model for CQ included an absorption lag. Weight was predictive of exposure for both AZ and CQ. Assuming equivalent dosing (mg/kg), AZ and CQ exposure in children would be expected to be lower than that in adults, suggesting that children may require a higher dose (mg/kg) than adults to achieve the same AZ and CQ exposure.

## Background

Worldwide, malaria remains a burden to global health. The World Health Organization reports that approximately 3.3 billion people were at risk of malaria in 2010, with the population of sub-Saharan Africa at the highest risk [1]. The combination of AZ and CQ exhibits synergistic activity against CQ-resistant strains of *Plasmodium falciparum in vitro* and *in vivo*[2-4]. Results from Phase 2 and 3 studies demonstrated that the combination of AZ and CQ had ~95% or more efficacy against symptomatic uncomplicated *P. falciparum* malaria in various geographic areas [3,5,6]. To be useful in combination, anti-malarial agents must not have clinically significant pharmacokinetic (PK) interactions. A lack of a PK interaction between AZ and CQ has been demonstrated [7]. In healthy adults, CQ did not have any clinically relevant effect on the PK of AZ; AZ also did not have any clinically relevant effect on either the PK of CQ or its active metabolite, desethylchloroquine (DECQ).

For adults, an investigational AZCQ tablet formulation has been developed containing AZ 250 mg and CQ 155 mg and has been evaluated in a bioavailability study in healthy adult volunteers with intensive PK sampling [8]. In this study, the AZCQ tablet was shown to be bioequivalent to the AZ 500 mg (Zithromax®, Pfizer Inc, New York, NY, USA) and CQ 300 mg (Aralen®, Sanofi Aventis, Bridgewater, NJ, USA) tablets taken individually in healthy adults using area under the concentration-time curve (AUC) as the criterion of exposure. Two additional paediatric AZCQ tablet formulations (AZ 300 mg/CQ 150 mg per tablet and AZ 150 mg/CQ 50 mg per tablet) were developed and evaluated in paediatric patients with malaria in Africa using sparse PK sampling.

The population PK analysis approach can be helpful in characterizing drug concentration-time profiles and between-subject variability in the PK. In addition, these analyses are useful to identify factors that are predictive of PK variability. Therefore, this population PK analysis was performed by combining the data from the bioavailability and paediatric treatment studies to evaluate the population PK of AZ and CQ in healthy adults from US and paediatric malaria subjects from sub-Saharan Africa.

## Methods

### Study design

PK data from two studies were pooled and analysed. The first study was an open-label efficacy study that compared the efficacy of AZCQ versus artemether-lumefantrine (AL) for the treatment of uncomplicated *P. falciparum* malaria in children in sub-Saharan Africa. In this study, patients were enrolled in two cohorts by age (Cohort 1 [5–12 years] and Cohort 2 [6–59 months]) and randomized to either AZCQ or AL treatment. This analysis only includes data from AZCQ-treated patients. In AZCQ-treated patients, dosing of AZCQ was based on weight, with patients receiving approximately 30 mg/kg of AZ and 10 mg/kg of CQ. AZCQ tablets were given once daily for three days based on weight (≥20 kg weight: AZ/CQ 300/100 mg strength tablet; 5 to <20 kg weight: AZ/CQ 150/50 mg strength tablet).

The second study was an open-label, randomized, single dose study to estimate the bioavailability of AZCQ tablets in healthy adult volunteers in the US. Subjects in this study were randomized to receive either two AZCQ tablets (AZ/CQ 250/155 mg strength tablet; test treatment) or co-administration of individual commercial tablets of AZ 500 mg and CQ 300 mg (reference treatment).

### Blood sampling and analysis

In the paediatric study, sparse blood samples were collected from both cohorts to determine AZ concentration in serum and CQ/desethylchloroquine (DECQ) concentration in plasma prior to AZCQ dosing (window: –1 to 0 h) on day 0 and day 2. On day 2, blood samples were also collected 3 h (window: 2–4 h) and 8 h (window: 6–10 h) after AZCQ dosing. In addition, blood samples were collected at a random time point on day 7. Blood samples in the bioavailability study were collected to assess serum AZ and plasma CQ concentrations at 0 (pre dose), 0.5, 1, 2, 3, 4, 6, 8, 12, 16, 24, 36, 48, 72 and 96 h post dose.

PK samples from both studies were analysed in the same bioanalytical laboratories (Centero Research in Houston, TX, USA for CQ and DECQ; Bioanalytical System Ltd in Warwickshire, UK for AZ). Plasma or serum samples were analysed for serum AZ, plasma CQ and plasma DECQ concentrations using validated, sensitive, specific high-performance liquid chromatography with tandem mass spectrometry assays [8]. The assays had a lower limit of quantification of 10.0, 1.0 and 0.50 ng/mL for AZ, CQ and DECQ, respectively. In the data analysis, observations that were below the limit of quantification were excluded from the database. The assay accuracy (expressed as % relative error: %RE) of the quality control samples used during the sample analysis ranged from 0% to 6.1% in the paediatric study and from -2.1% to 4.7% in the adult study for AZ, from -3.2 to 9.8% in the paediatric study and from -0.4% to 3% in the adult study for CQ, and from 0.0% to 14.5% in the paediatric study for DECQ. The assay precision data were ≤ 4.9%CV in the paediatric study and ≤4.7% in the adult study for AZ, ≤11.2% CV in the paediatric study and ≤8.9% in the adult study for CQ, and ≤10.8% in the paediatric study for DECQ.

### Population PK analysis

Concentration-time data collected in both studies were analysed using the mixed-effects modelling tool, NONMEM® (version 7, level 2; ICON Plc, Dublin, Ireland). All clearance and distribution parameters are reported as the ratio of the parameter and bioavailability since oral dosing was not accompanied by an intravenous dose. All evaluations used a log-transform both sides approach.

Population PK models were constructed to evaluate the concentration-time data for AZ and CQ and to identify any covariates predictive of PK behaviour using standard approaches. The structural model consisted of a compartmental PK model to describe the observed concentration-time profiles as well as between subject variability in the PK behaviour. For both AZ and CQ, two- and three-compartment models were evaluated. In addition, a one-compartment model was also evaluated for completeness, but did not describe the data well and was therefore not used for either drug.

The covariate models in this PK model were defined to represent the shift in value of the parameter of interest to a value for a hypothetical patient. The reference patient for this analysis was 40 years of age, weighed 70 kg and had a body surface area of 1.73 m^2^ and a body mass index of 15 kg/m^2^. Covariates examined as potential predictors of PK activity are listed in Table 1. Only those covariates that individually influenced the structural parameters were added to create the full model. The full model then underwent reduction where covariates were removed one at a time and the impact of removal was assessed (backward deletion method). The covariate was retained in the final model if its removal resulted in an increase in the objective function of ≥10.83 points (p < 0.001) from the full model.

**Table 1:** Covariates assessed in the pharmacokinetic analysis

**Covariate (unit)**	**Type**
Actual dose (mg)	Continuous
Study (1 or 2)	Categorical
Age (years)	Continuous
Gender	Categorical
Race	Categorical
Weight at baseline (kg)	Continuous
Height (cm)	Continuous
Body surface area at baseline (m^2^)	Continuous
Body mass index at baseline (kg/m^2^)	Continuous
Ideal body weight at baseline (kg)	Continuous
Lean body weight at baseline (kg)	Continuous
Aspartate aminotransferase at baseline (U/L)	Continuous
Alanine aminotransferase at baseline (U/L)	Continuous
Bilirubin at baseline (mg/dL)	Continuous
Albumin at baseline (g/dL)	Continuous
Calculated creatinine clearance at baseline (mL/min)	Continuous

Two different allometric models using weight normalized PK parameters to size were evaluated [9]. The first model used an allometric coefficient of 0.75 for clearances and 1 for volumes. The second model used estimated allometric coefficients for clearances and volumes. Wherever possible, symmetric 95% confidence intervals were computed using the asymptotic standard errors of the parameter estimates.

## Results

### Subject disposition and demographics

The final dataset consisted of 1,198 and 1,197 evaluable measurable AZ and CQ concentration observations from 219 subjects including 40 adults, 123 paediatric subjects aged < 5 years and 56 paediatric subjects aged 5 to 12 years.

### Models

The final model for AZ in serum following AZCQ administration was a three-compartment model with linear clearance and absorption and no absorption lag time. The final model for CQ in plasma was a two-compartment model with linear clearance and an absorption lag time. Both models included actual body weight with fixed allometric scaling. In the AZ model, shrinkage was acceptable (defined as ≤20%) for clearance (CL/F), but was high (51.8%) for central compartment volume of distribution (V1/F) that is considered likely due to the sparse sampling in the paediatric study. In the CQ model, shrinkage was acceptable for CL/F and V1/F, but was elevated for the absorption rate constant (62%) that could be due to the sparse sampling in the paediatric study. The condition number for the base model was less than 20 (AZ: 13.4; CQ: 5.3) for both AZ and CQ suggesting no notable co-linearity. Thus, covariate evaluations on CL/F were considered appropriate.

DECQ only had sparse concentration data from the paediatric patients and no data from the adult bioavailability study were available; thus, a population PK model for DECQ could not be identified.

### PK parameters

The parameters for the final AZ and CQ population PK models are provided in Table 2. Body weight was found to be influential for both AZ and CQ pharmacokinetics.

**Table 2:** Parameter estimates for final population PK model: azithromycin in serum and chloroquine in plasma

**Parameter (units)**	**PK parameter mean (%RSE)**	**Inter-individual variability %CV (%RSE)**	**PK parameter mean (%RSE)**	**Inter-individual variability %CV (%RSE)**
	**Azithromycin**		**Chloroquine**	
CL/F (L/h)	100 (6.1)	31.3 (17.6)	59.1 (2.3)	30.5 (12.2)
Normalized for weight	0.75 FIXED		0.75 FIXED	
V1/F (L)	186 (22.4)	113 (31.8)	2,870 (4.3)	46.6 (15.7)
Normalized for weight	1 FIXED	CL:V1 covariance 0.28	1 FIXED	CL:V1 covariance 0.72
Ka (1/h)	0.259 (10.2)	NE	6.12 (29.4)	NE
Q2/F (L/h)	180 (8.2)	NE	61.4 (11.3)	NE
Normalized for weight	0.75 FIXED		0.75 FIXED	
V2/F (L)	2,890 (4.8)	NE	1,890 (4.9)	NE
Normalized for weight	1 FIXED		1 FIXED	
LAG (h)	NE	NE	0.387 (6.7)	NE
Q3/F (L/h)	10.6 (19.0)	NE	NE	NE
V3/F (L)	2,610 (46.7)	NE	NE	NE
Residual error (%RSE)	0.406 (2.4)	NE	0.249 (2.6)	NE

%CV, Percent coefficient of variation; %RSE, Relative standard error expressed as percentage; CL/F, Clearance normalized for bioavailability; IIV, Inter-individual variability; Ka, Absorption rate constant; LAG, Lag time; NE, Not estimated; PK, Pharmacokinetics; Q2/F, Inter-compartmental clearance from central to first peripheral compartment; Q3/F, Inter-compartmental clearance from central to second peripheral compartment; V1/F, Volume of distribution of the central compartment; V2/F, Volume of distribution of first peripheral compartment; V3/F, Volume of distribution of second peripheral compartment.

**Figure 1:**
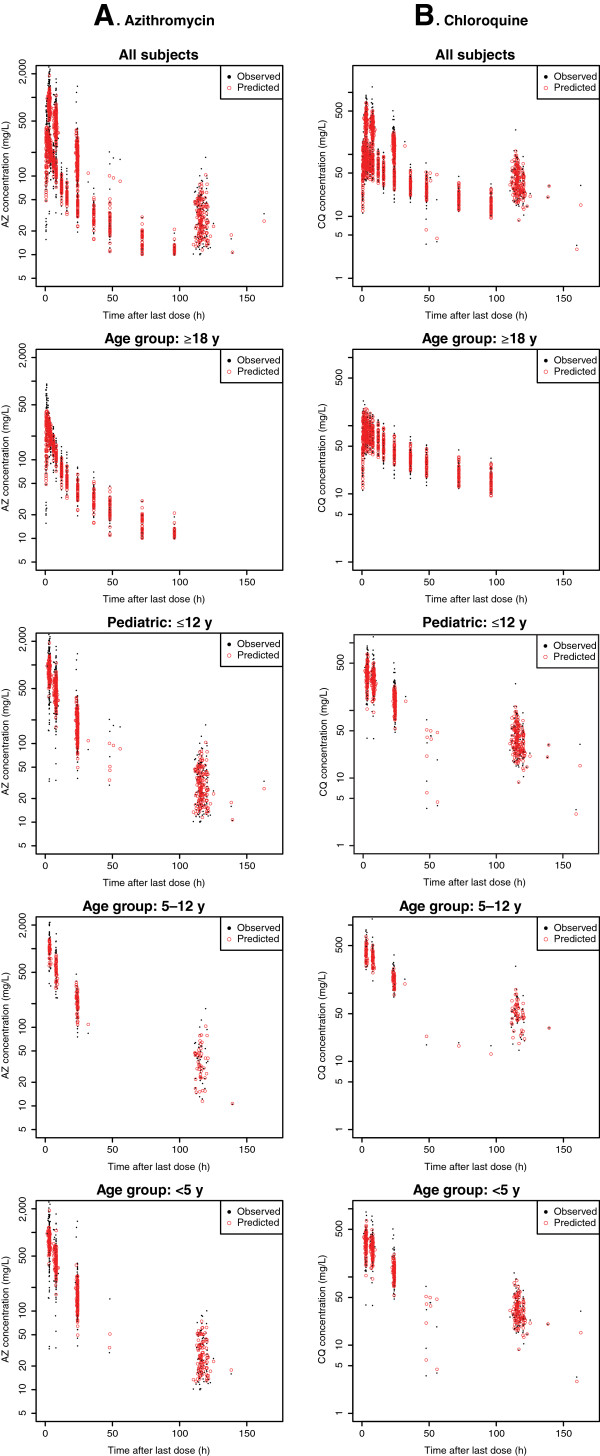
**Final population pharmacokinetic (PK) model: predicted and observed (A) serum azithromycin (AZ) and (B) plasma chloroquine (CQ) concentrations versus time after last dose relative to sampling time.** Figure 1 shows **(A)** serum AZ and **(B)** plasma CQ measured and predicted concentrations in various age groups. Black filled circles represent observed data points and red open circles are population predicted values for the final PK model. Good concordance between observed and predicted data points is evident.

**Figure 2:**
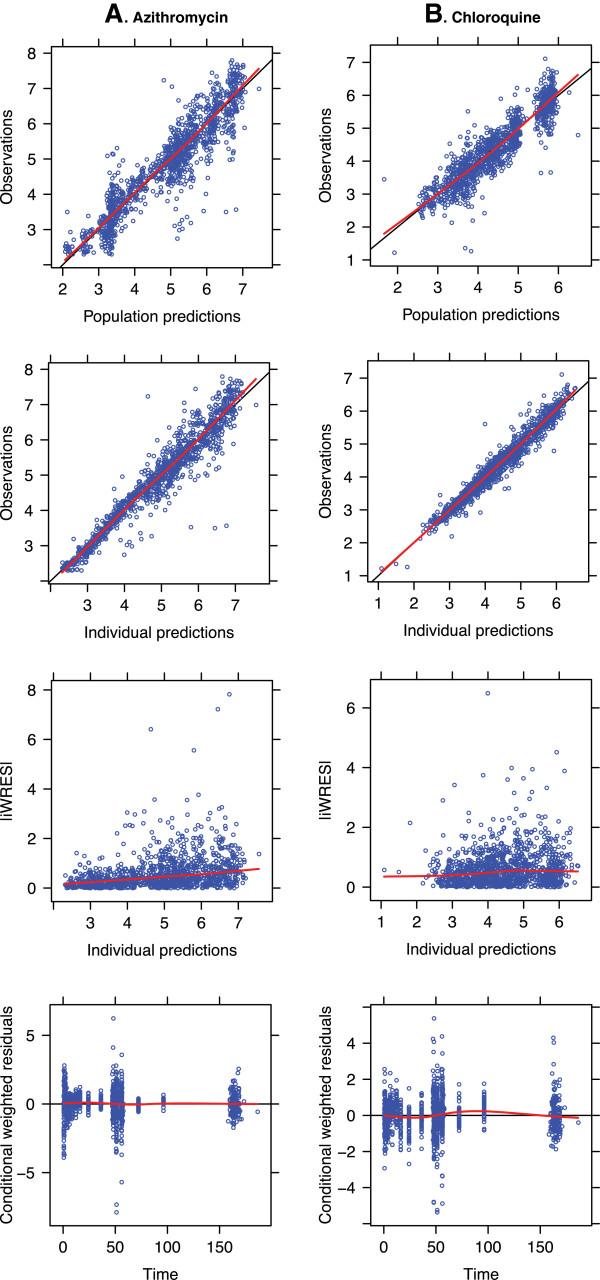
**Goodness of fit plots for all ages for the final (A) serum azithromycin (AZ) and (B) plasma chloroquine (CQ) pharmacokinetic models.** Figure 2 shows goodness of fit plots for **(A)** serum AZ and **(B)** plasma CQ measured and predicted concentrations. No systematic bias is evident and good concordance between observed and predicted concentrations is evident in the aggregate data.

**Figure 3:**
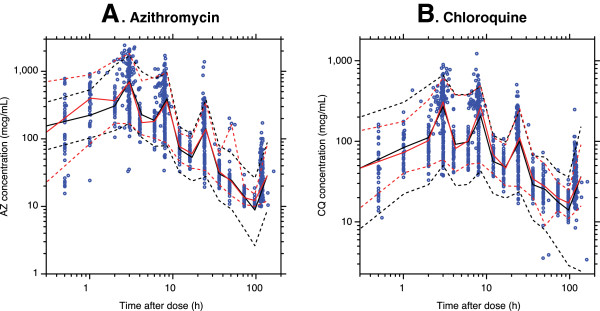
**Visual predictive check for all concentration-time data for (A) serum azithromycin (AZ) and (B) plasma chloroquine (CQ).** Figure 3 shows the log-log plot of the visual predictive check for the final pharmacokinetic model using all data in the dataset. Open blue circles are observed concentrations. The solid black line represents the median of the predicted data (black dashed lines represent the 95% confidence interval [CI]). The solid red line represents the median of the actual data (red dashed lines represent the 95% CI). No apparent bias was noted in the final model and overall performance was considered adequate.

Good concordance was noted between the values predicted from the PK model versus the observed concentrations of AZ and CQ (Figure 1). Goodness of fit plots also indicated that the PK models adequately described the PK data for both AZ and CQ and did not suggest substantial bias (Figure 2). A visual predicted check from all the data in the final model indicated that, for both AZ and CQ, the majority of observed concentrations fell within the predicted interquartile ranges (Figure 3).

There was no overall bias or evidence of substantial model misspecification for either AZ or CQ. Body weight as an allometric model was the only covariate in the final AZ and CQ PK models.

**Figure 4:**
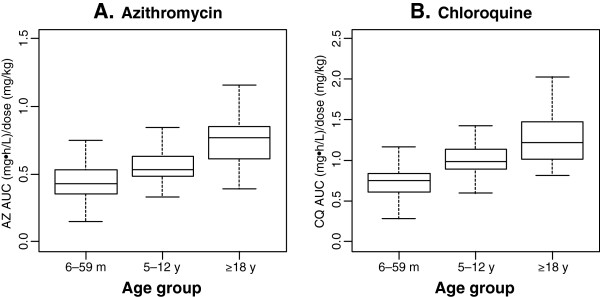
**Comparison of dose-normalized area under the concentration-time curve (AUC**_**inf**_**) by age group for (A) serum azithromycin (AZ) and (B) plasma chloroquine (CQ).**

The dose-normalized AUC values from time 0 to infinity (AUC_inf_) of AZ and CQ were estimated from the respective administered doses and the individual subject clearances (CL/F) then normalized by the administered dose divided by body weight (AUC_inf_/dose = dose/CL/[dose/weight]). The paediatric population had significantly lower values than that of the adult population in the dose (mg/kg)-normalized AUC_inf_ for both AZ (0.488 vs 0.745 mg*h/L/(mg/kg), p < 0.00001) and CQ (0.836 vs 1.27 mg*h/L/(mg/kg), p < 0.00001), indicating a lower (L/h/kg) clearance value in adults as compared to paediatric subjects. Comparison of dose-normalized AUC_inf_ for both AZ and CQ by age group is graphically presented in Figure 4.

## Discussion and conclusions

AZ PK and CQ PK were well described using a three- and two-compartment model, respectively. These models were in general agreement with models previously published [10-13]. In the models of the current study, both AZ and CQ exhibited linear absorption and clearance in children and adults, but CQ showed an absorption lag. Due to the sparse PK sampling in the paediatric study, it would be difficult to identify such an absorption delay in the appearance of drug in the paediatric patients, and the ability to identify if an absorption phase exists because of the intensive sampling in the adult study. Consequently, it is not possible to determine if the lag time is the same in the paediatric patients as in the adults. Previously published values for AZ PK parameters were generally consistent with those estimated in the present analysis [10,12]. For CQ, other studies have found that a two-compartment model best fit data in women [11] and children [13]. However, neither of these models included an absorption lag or transit.

A positive correlation between age and CQ clearance was reported by Obua *et al*. [13], but was not confirmed in this analysis, although weight and age are highly positively correlated in paediatric subjects. However, weight was predictive of exposure for both AZ and CQ in this analysis.

Drug clearance in children was higher than that in adults when evaluated on a weight-normalized basis. Allometric models have been developed to address the higher clearance (L/h/kg) observed in children [14,15]. Assuming equivalent dosing (mg/kg), AZ and CQ exposure in children would be expected to be lower than that in adults, suggesting that children may require a higher dose (mg/kg) than adults to achieve the same AZ and CQ exposure.

In conclusion, pharmacokinetics for AZ and CQ was well described using a three-compartment model and two-compartment model, respectively, in both healthy adult subjects and paediatric patients with symptomatic uncomplicated falciparum malaria following the full three-day course of AZCQ treatment. Both AZ and CQ exhibited linear absorption and linear clearance. In addition, there was an absorption lag in CQ. The effect of body size on PK was described using an allometric function based on normalized weight on primary AZ and CQ PK parameters. Drug clearance (L/h/kg) in children was higher than that in adults when evaluated on a weight-normalized basis. This suggests that exposure (AUC) of AZ and CQ in the paediatric patients would be expected to be lower than that experienced by the adults with equal dosing on a mg/kg basis and higher mg/kg dosing may be required in this population.

## Abbreviations

%CV: Percent coefficient of variation; %RSE: Relative standard error expressed as percentage; AL: Artemether-lumefantrine; AUC: Area under the concentration-time curve; AUCinf: Area under the concentration-time curve from time 0 to time infinity; AZ: Azithromycin; AZCQ: Fixed-dose combination tablet treatment of azithromycin and chloroquine; CL/F: Clearance; Cmax: Maximum concentration; CQ: Chloroquine; DECQ: Desethylchloroquine; IIV: Inter-individual variability; Ka: Absorption rate constant; LAG: Lag time; NE: Not estimated; PK: Pharmacokinetic; Q2/F: Inter-compartmental clearance from central to first peripheral compartment; Q3/F: Inter-compartmental clearance from central to second peripheral compartment; Tmax: Time to maximum concentration; V1/F: Volume of distribution; V2/F: Volume of distribution of first peripheral compartment; V3/F: Volume of distribution of second peripheral compartment.

## Competing interests

QZ and TT are full-time employees of and own stock in Pfizer Inc. RC was a full time employee of Pfizer Inc when these studies were conducted and owns shares in Pfizer Inc. DRM is a full-time employee of Projections Research, Inc.

## Authors’ contributions

All authors contributed to the development and conduct of the analysis. All authors reviewed and revised manuscript outlines and drafts and approved the final version.
